# Metamizole-Associated Adverse Events: A Systematic Review and Meta-Analysis

**DOI:** 10.1371/journal.pone.0122918

**Published:** 2015-04-13

**Authors:** Thomas Kötter, Bruno R. da Costa, Margrit Fässler, Eva Blozik, Klaus Linde, Peter Jüni, Stephan Reichenbach, Martin Scherer

**Affiliations:** 1 Institute of Social Medicine and Epidemiology, University of Lübeck, Lübeck, Germany; 2 Department of Primary Medical Care, University Medical Center Hamburg-Eppendorf, Hamburg, Germany; 3 Institute of Primary Health Care (BIHAM), University of Bern, Bern, Switzerland; 4 Institute of General Practice, Technische Universität München, München, Germany; 5 Institute of Biomedical Ethics, University of Zürich, Zürich, Switzerland; 6 Institute of Social and Preventive Medicine, University of Bern, Bern, Switzerland; 7 Bern University Hospital, Bern, Switzerland; University of Chieti, ITALY

## Abstract

**Background:**

Metamizole is used to treat pain in many parts of the world. Information on the safety profile of metamizole is scarce; no conclusive summary of the literature exists.

**Objective:**

To determine whether metamizole is clinically safe compared to placebo and other analgesics.

**Methods:**

We searched CENTRAL, MEDLINE, EMBASE, CINAHL, and several clinical trial registries. We screened the reference lists of included trials and previous systematic reviews. We included randomized controlled trials that compared the effects of metamizole, administered to adults in any form and for any indication, to other analgesics or to placebo. Two authors extracted data regarding trial design and size, indications for pain medication, patient characteristics, treatment regimens, and methodological characteristics. Adverse events (AEs), serious adverse events (SAEs), and dropouts were assessed. We conducted separate meta-analyses for each metamizole comparator, using standard inverse-variance random effects meta-analysis to pool the estimates across trials, reported as risk ratios (RRs). We calculated the DerSimonian and Laird variance estimate T^2^ to measure heterogeneity between trials. The pre-specified primary end point was any AE during the trial period.

**Results:**

Of the 696 potentially eligible trials, 79 trials including almost 4000 patients with short-term metamizole use of less than two weeks met our inclusion criteria. Fewer AEs were reported for metamizole compared to opioids, RR = 0.79 (confidence interval 0.79 to 0.96). We found no differences between metamizole and placebo, paracetamol and NSAIDs. Only a few SAEs were reported, with no difference between metamizole and other analgesics. No agranulocytosis or deaths were reported. Our results were limited by the mediocre overall quality of the reports.

**Conclusion:**

For short-term use in the hospital setting, metamizole seems to be a safe choice when compared to other widely used analgesics. High-quality, adequately sized trials assessing the intermediate- and long-term safety of metamizole are needed.

## Introduction

Metamizole, or dipyrone, is a pyrazolone derivate whose structure is closely related to that of amidopyrine. It was launched commercially as an analgesic and antipyretic by Hoechst AG in 1922 [1], and is commonly used to treat postoperative pain, colic pain, cancer pain and migraine [2–4]. In many parts of the world, including most countries in the European Union and Latin America, it is the most popular non-opioid first-line analgesic and is sometimes even available over-the-counter. A few countries however, including the United States, the United Kingdom, Sweden, and most recently India [5], have banned metamizole because health authorities judged the risk of agranulocytosis to outweigh the benefits [6–8].

Although used for more than 90 years, the risks and harms of metamizole are not well documented, and information on adverse events related to metamizole is scarce. There are no large randomized controlled trials or conclusive summaries of the existing literature [9]. Three current Cochrane reviews on the effectiveness and safety of metamizole for acute postoperative pain [2], acute renal colic pain [3], and acute primary headaches [4] concluded that metamizole offers good short-term pain relief. In each of these systematic reviews, however, the number of included participants was too small and the authors did not conduct a meta-analysis of safety issues, but they did associate metamizole with somnolence, gastric discomfort and nausea.

Given the still ongoing controversies about the gastrointestinal and cardiovascular safety profiles of non-steroidal anti-inflammatory drugs (NSAIDs) [10–12], which are among the alternatives to metamizole, a comprehensive systematic review of randomized controlled trials investigating metamizole use for all indications is needed. We therefore determined whether metamizole was clinically safe compared to placebo and other commonly used analgesics.

## Methods

We used a standard review protocol that was submitted to the funding body prior to the start of the study.

### Literature Search

We searched electronic databases from inception to February 2013, without language restriction, including the Cochrane Controlled Trials Register (CENTRAL) through The Cochrane Library (http://mrw.interscience.wiley.com/cochrane/), MEDLINE and EMBASE through the Ovid platform (http://www.ovid.com/) and CINAHL through EBSCOhost. The search algorithm for EMBASE is displayed in S1 Table (it was slightly modified for the other databases). We searched several clinical trial registries (http://www.controlled-trials.com/;http://www.clinicaltrials.gov/, http://www.actr.org.au/, http://www.umin.ac.jp/ctr/) to identify unpublished trials. We also screened the reference lists of included trials and previous systematic reviews.

### Trial selection

We included randomized controlled trials that compared the effects of metamizole in adult patients, administered in any form and for any indication, to other analgesics or to placebo. Quasi-randomized trials were not eligible. We selected for inclusion trials that reported any adverse events. We excluded trials in which metamizole was a co-treatment in more than one arm.

Two review authors independently evaluated all titles and abstracts for eligibility. We resolved disagreements by consensus or discussion with a third reviewer. We included studies regardless of length of follow-up or language of publication. If multiple reports described the same trial, we chose the most recent full-text publication in a peer-reviewed journal as the main report.

### Outcome measures

The pre-specified primary end point was any adverse event during the trial period. Secondary end points were serious adverse events, overall dropouts and dropouts due to adverse events and serious adverse events. We defined serious adverse events as those that resulted in inpatient hospitalization, persistent or significant disability, congenital abnormality of offspring, life-threatening events or death [13]. We extracted the number of patients per group who experienced at least one event before the end of the trial, and categorized and reported them using the International Classification of Primary Care (ICPC; 2^nd^ edition), which allows for reporting of both complaints and diagnosis [14].

### Data Collection and Quality Assessment

Two authors independently extracted data from the full-text articles. They used a standardized, piloted extraction form accompanied by a codebook created for this review [15]. Reviewers resolved disagreements by consensus or through discussion with a third author.

We extracted data regarding trial size, trial design, trial duration (defined as time from first application until end of follow-up), indication for pain medication, patient characteristics (sex, age), treatment regimens (application form, duration and/or frequency of treatment), adverse events and funding source. We extracted data only from the first period of crossover trials, because carry-over effects might be present in the later periods. We attempted to contact studies’ corresponding authors to obtain missing information.

Two review authors independently assessed concealment of treatment allocation, blinding of patients, adequate adverse event assessment and adequacy of analyses. We considered allocation concealment to be adequate if the investigators who selected patients were unable to guess which treatment patients would be allocated to. We considered patients to be blinded if the trial was reported as “double blind”. Adverse event assessment was considered adequate if it was conducted systematically and prospectively. Analyses were considered adequate if all recruited patients were analyzed in the group to which they were originally allocated, regardless of the treatment received (intention-to-treat principle). See S2 Table for definitions used to classify trials according to components of methodological quality. Disagreements were resolved by discussion with a third reviewer and subsequent consensus.

### Data Synthesis and Analysis

We conducted separate meta-analyses for each metamizole comparator. We first calculated the log risk ratio and its standard error for each trial, and then used standard inverse-variance random effects meta-analysis to pool these estimates across trials [16]. The pooled estimate was then exponentiated to report treatment effect estimates as risk ratios (RRs). A RR below 1 indicates that metamizole is a safer intervention than its comparator. We calculated the DerSimonian and Laird variance estimate T^2^ to measure heterogeneity between trials [16]. A T^2^ of 0.04 was pre-specified to represent low heterogeneity, 0.09 moderate heterogeneity and 0.16 high heterogeneity between trials [17]. We performed stratified analyses of primary and secondary outcomes according to dose and route of administration. Uni-variable random-effects meta-regression models were used for tests of interaction between outcomes and these characteristics. We distinguished between single dose and multiple doses, determined the association of the log risk ratio with daily dose and cumulative dose on a continuous scale, and classified route of administration as i.m., i.v., or p.o. for this purpose. All p-values are two-sided. Analyses were conducted using STATA, release 11 (Stata-Corp, College Station, Texas).

## Results

**Fig 1:**
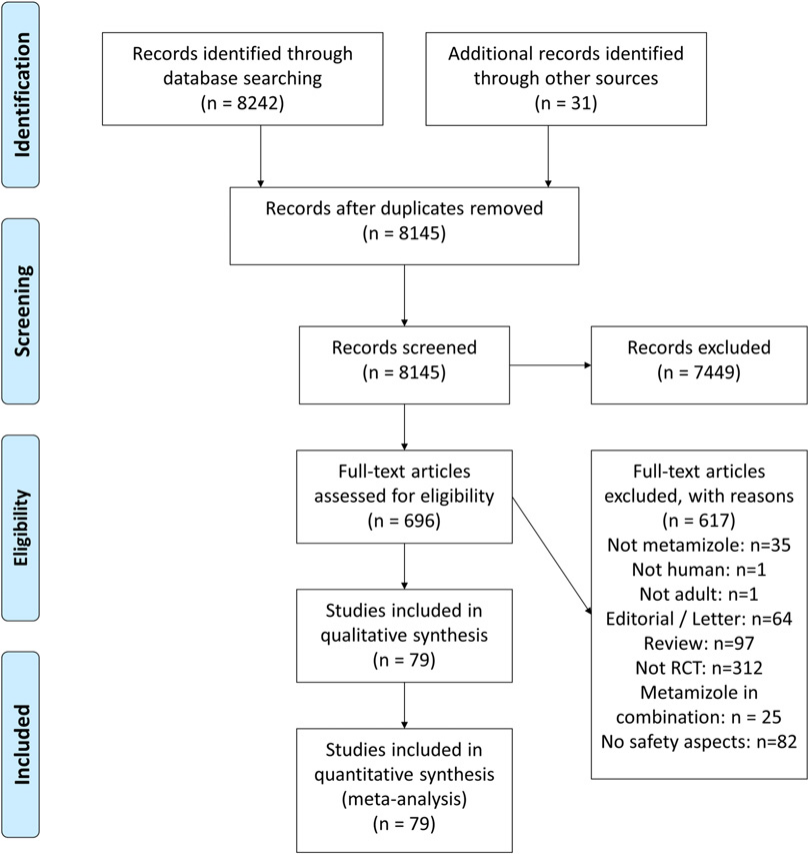
PRISMA flowchart. The 79 randomized controlled trials [18–95] assessed mainly short-term use of common analgesics. Forty-two trials (53%) investigated the use of only a single dose of metamizole. In addition, 18 trials (23%) had a maximum treatment duration of only one day, while in 17 trials treatment lasted from two days up to one week (22%) and only two trials (2%) had a treatment duration of two weeks. Average length of follow-up ranged from 0.33 to 366 hours (median: 24 hours). Four trials (5%) had sample sizes of at least 100 patients per randomized group (median: 30 patients).

We identified 8242 references in our literature search, of which 696 were potentially eligible. Seventy-nine reports describing 79 randomized controlled trials met our inclusion criteria and were included in the meta-analysis (see PRISMA (Preferred Reporting Items for Systematic Reviews and Meta-Analyses) flowchart [Fig 1] and checklist [S1 PRISMA Checklist]). Forty-nine trials were published in English, 12 in Spanish, six in Portuguese, six in German, four in Italian, and two in Turkish. Trials were conducted in several European and Latin American countries where metamizole is widely available. All trials were published as full-text journal articles. The median year of publication was 1995 (range: 1974–2011). A search of trial registries yielded no ongoing trials.

The average age of participants ranged from 22–64 years (median: 45 years). Women made up 45.8% of the subjects. A total of 3716 patients received metamizole, 1077 received placebo, 303 received aspirin, 1983 received NSAIDs, 829 received opioids, 362 received paracetamol, and 156 patients received other pain medications. Very few of the 79 included randomized controlled trials were conducted in the ambulatory setting. Table 1 describes the clinical characteristics of the trials.

**Table 1:** Clinical characteristics of the trials.

Trial	year	n^1^	Indication	Comparison	form of application^2^	Treatment duration (days)
**Ajgaonkar**	1985	42/46	Fever	Placebo	p.o.	single dose
**Arnau**	1991	217/ 116	Renal colic	Diclofenac, Pethidine	i.m.	single dose
**Atalay**	1995	14/38	Post-OP	Fentanyl, Morphine	i.m. / epidural catheter	single dose
**Babej-Dölle**	1994	88/ 172		Diclofenac, Placebo	i.m.	2
**Bagan**	1998	40/80	Post-OP	Dexketoprofen	p.o.	3
**Bigal**	2002	74/60	Migraine	Placebo	i.v.	single dose
**Bilgin**	2003	25/25	Post-OP	Bupivacaine	extrapleural catheter	3
**Blendinger**	1980	7/10	Post-OP	ASS	i.v.	single dose
**Bloch**	1985	68/65	Post-OP	Pethidine	i.m.	2
**Boraks**	1987	39/ 120	Post-OP	Flurbiprofen, Placebo	p.o.	single dose
**Braun**	1999	76/79	Post-OP	Placebo	i.v.	0.042
**Brodner**	2011	49/ 147	Post-OP	Parecoxib, PCM, Placebo	i.v.	2
**Castro**	2000	30/68	Post-OP	Ketorolac, Tramadol	i.v.	1
**Castro Gonzales**	1986	28/58	Post-OP	Dexketoprofen, Ibuprofen	p.o.	1.667
**Cruz**	2002	30/30	Fever	Propacetamol	i.v.	single dose
**De Miguel Rivero**	1997	35/72	Post-OP	Ibuprofen, Placebo	i.m. / p.o.	single dose
**Diaz-Chavez**	2009	47/48	Post-OP	Ketorolac	i.v. / p.o.	1
**Dos Santos Pereira**	1986	28/57	Post-OP	Acetaminophen, Placebo	p.o.	single dose
**Duarte Souza**	2007	18/16	Cancer	Placebo	p.o.	2
**Fernandes Filho**	2006	12/15	Migraine	MCP	i.v.	single dose
**Ferrario**	1984	14/14	Post-OP	ASS	i.m. / i.v.	single dose
**Gomes-Marquez**	2004	25/25	Post-OP	Parecoxib	i.v.	single dose
**Gonzalez-Garcia**	1994	unclear	Post-OP	Ketorolac	p.o.	single dose
**Grundmann**	2006	20/60	Post-OP	Parecoxib, PCM, Placebo	i.v.	single dose
**Guberti**	1982	14/14	Post-OP	Nefopam	i.v.	4
**Hernandez Llenas**	1997	15/15	Post-OP	Diclofenac	i.v.	0.336
**Herrera Barroso**	1982	30/60	Post-OP	Zomepirac, Placebo	p.o.	single dose
**Ibarra-Ibarra**	1993	48/49	Post-OP	Ketorolac	i.m.	single dose
**Jage**	1990	40/40	Post-OP	Placebo	i.v.	1
**Jovic**	2008	30/30	Post-OP	Ketoprofen	i.v.	5
**Kampe**	2006	20/20	Post-OP	PCM	i.v.	1
**Karaman**	2010	30/60	Post-OP	Dexketoprofen, PCM	i.v.	1
**Kemal**	2007	20/20	Post-OP	Lornoxicam	i.v.	1
**Knüsel**	1982	40/40	Osteoarthritis	Zomepirac	p.o.	14
**Krymchantowski**	2008	15/15	Migraine	Lysin Clonixinate	i.v.	single dose
**Landwehr**	2005	13/12	Post-OP	PCM	i.v.	1.021
**Lehmann**	2001	40/40	Post-OP	Placebo	i.v.	1
**Lehtonen**	1983	45/124	Renal colic	Indomethacin, Pethidine	i.v.	single dose
**Marin-Bertolin**	1997	46/46	Post-OP	Ketorolac	i.m.	2
**Martin-Duce**	1997	187/48	Post-OP	Diclofenac	i.m. / i.v.	single dose
**Martinez-Martin**	2001	204/207	Migraine	ASS	p.o.	single dose
**Mateu**	1992	38/80	Post-Trauma	ASS, PCM	p.o.	single dose
**Mehta**	1986	91/163	Post-OP	ASS, Placebo	p.o.	single dose
**Monso**	1996	37/67	Shivering	Pethidine, Placebo	i.v.	single dose
**Muriel**	1993	87/41	Renal colic	Diclofenac	i.v.	single dose
**Muriel-Villoria**	1995	239/54	Renal colic	Diclofenac	i.m.	single dose
**Ocampo Flores**	1986	15/30	Post-OP	Ibuprofen, Dextropropoxifene	p.o.	1.667
**Pardo**	1984	30/60	Renal colic	Ceruletide, Placebo	i.v.	single dose
**Patel**	1980	51/49	Post-OP	Pethidine	i.m.	single dose
**Pavlik**	2004	32/32	Renal colic	Cizolirtin	i.v.	single dose
**Peiró**	2008	8/8	Pancreatitis	Morphine	i.v. / s.c.	2
**Pernia**	2000	30/37	Post-OP	Propacetamol	i.v.	1
**Pinto**	1984	27/29	Post-OP	Acetaminophen	p.o.	single dose
**Planas**	1998	147/106	Post-OP	Ibuprofen, Placebo	p.o.	single dose
**Prada**	1974	20/40	Post-OP	Pentazocine	supp.	single dose
**Primus**	1989	30/30	Renal colic	Tramadol	i.v.	single dose
**Rawal**	2001	40/80	Post-OP	Tramadol	p.o.	2
**Rejman**	1984	25/25	Biliary colic	Indomethacin	i.v.	single dose
**Reyes**	1988	25/25	Post-OP	Diclofenac	i.v.	single dose
**Reyes-Armijo**	1974	15/15	Migraine	Vitamin B1, B6	p.o.	14
**Rodriguez**	1994	79/42	Cancer	Morphine	p.o.	7
**Rubinstein**	1986	30/60	Post-OP	Acetaminophen	p.o.	single dose
**Sanchez-Carpena**	2003	108/225	Renal colic	Dexketoprofen	i.m.	single dose
**Sanchez-Carpena**	2007	103/205	Renal colic	Dexketoprofen	i.v.	single dose
**Saray**	2001	80/80	Post-OP	Diclofenac	i.m.	2
**Savoca**	1985	15/15	Post-OP	Imidazol-2-Hydroxybenzoat	p.o.	1
**Schmieder**	1993	25/49	Biliary colic	Butylscopolamin	i.v.	single dose
**Sener**	2008	40/160	Post-OP	Diclofenac, Ketoprofen, Lornoxicam, Placebo	i.m.	0.667
**Spacek**	2003	30/30	Post-OP	Placebo	i.v.	1
**Stankov**	1994	36/68	Renal colic	Butylscopolamin	i.v.	single dose
**Stankov**	1995	51/49	Post-OP	Tramadol	i.v.	single dose
**Steffen**	1997	20/20	Post-OP	Tramadol	i.v.	0.5
**Striebel**	1992	30/30	Post-OP	Placebo	i.v.	0.167
**Tempel**	1996	54/52	Post-OP	Placebo	i.v.	0.667
**Tonolli Jacob**	1986	33/66	Post-Trauma	Diclofenac, Placebo	i.m.	single dose
**Torres**	1993	50/100	Post-OP	Buprenorphin, Morphine	i.v.	0.072
**Torres**	2001	73/78	Post-OP	Tramadol	i.v.	1
**Uzun**	2010	23/20	Post-OP	Placebo	i.v.	single dose
**Vargha von Szeged**	1986	30/30	Migraine	Suprofen	p.o.	5

^1^: n (patients) Metamizole / n (patients) comparisons.

^2^: i.m.: intramuscular, i.v.: intravenous, p.o.: per os, supp: suppository, s.c.: subcutaneous.

Twenty-four studies (31%) used adequate randomization methods, fourteen studies (18%) adequately concealed allocation, fifty-five studies (70%) adequately blinded patients and 13 studies (17%) adequately assessed adverse events. Forty-two studies (53%) analyzed all patients according to the intention-to-treat principle. S3 Table describes the trials’ methodological characteristics. The heterogeneity between the studies was low for most adverse events (T^2^ <0.01). The data extracted from single studies can be found in S4 Table and S1 Dataset.

### Metamizole versus Placebo

**Fig 2:**
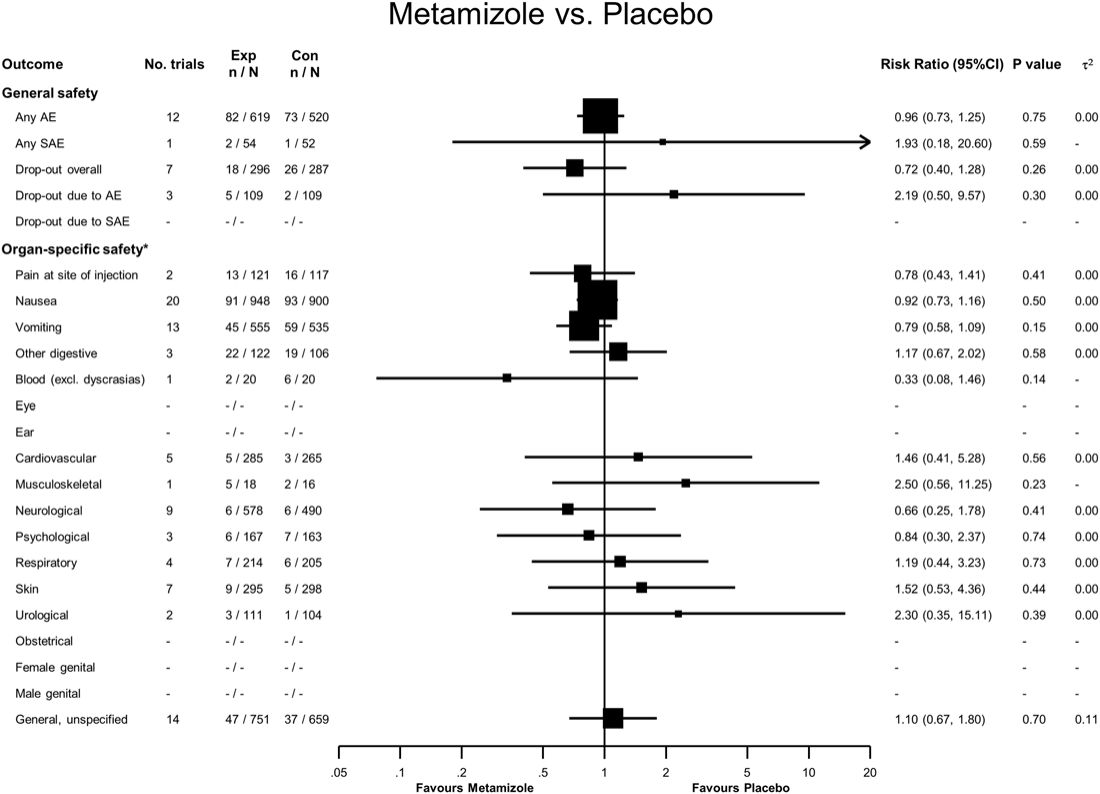
Forest plot—Metamizole versus Placebo. Categories according to the International Classification of Primary Care (see Methods section and [13]). Results from single studies are of limited interpretability but are displayed for the sake of completeness. RR = risk ratio. AE = adverse events. SAE = serious adverse events.

Eighty-two adverse events were reported in 619 patients treated with metamizole, compared to 73 adverse events in 520 patients treated with placebo, yielding a RR of 0.96 (95% confidence interval (CI) 0.73 to 1.25, see Fig 2). Two serious adverse events were reported in the metamizole group: one case of leucopenia due to septicemia following aspiration and one case of post-operative hemorrhage after prostatectomy. In comparison, one serious adverse event was reported in the placebo group, a case of leucopenia due to anastomosis insufficiency [90], yielding a RR of 1.93 (95% CI 0.18 to 20.6). For overall dropouts and dropouts due to adverse events, 95% CI overlapped the line of no difference. We found no statistically significant difference in organ-specific safety.

### Metamizole versus Paracetamol

**Fig 3:**
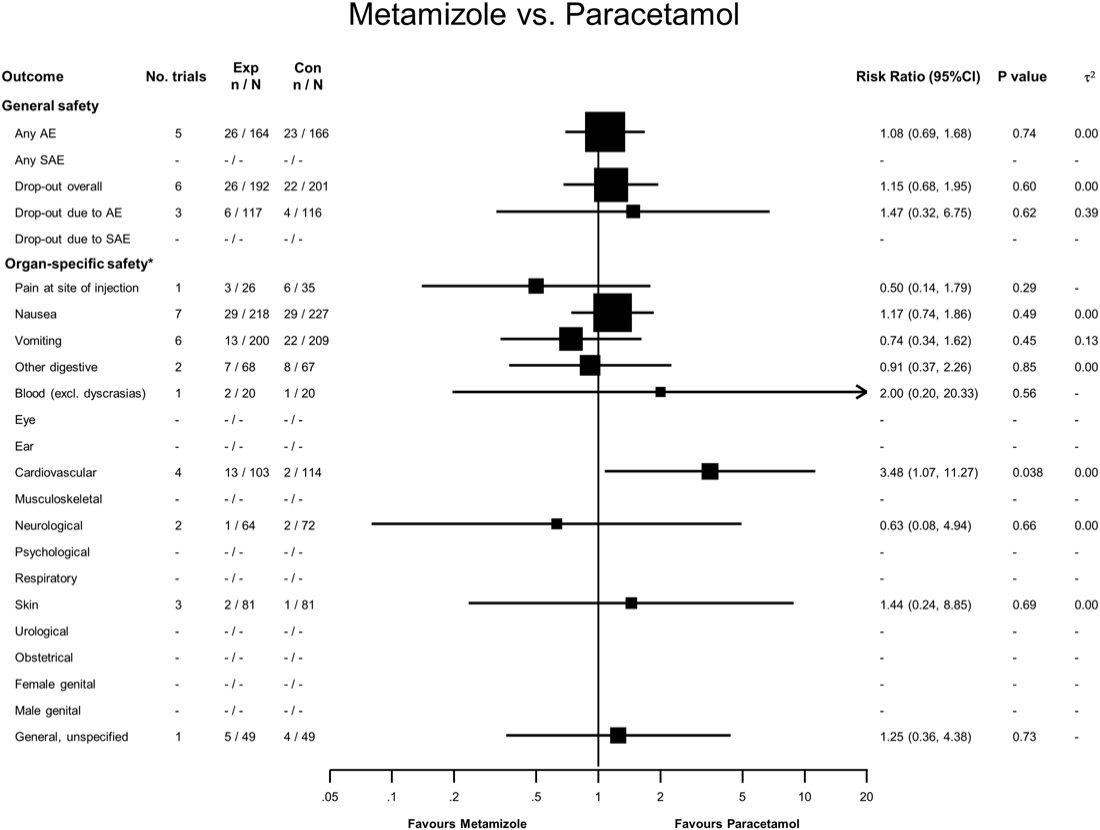
Forest plot—Metamizole versus Paracetamol. Categories according to the International Classification of Primary Care (see Methods section and [13]). Results from single studies are of limited interpretability but are displayed for the sake of completeness. RR = risk ratio. AE = adverse events. SAE = serious adverse events.

Twenty-six adverse events were reported in 164 patients treated with metamizole, and 23 in 166 treated with paracetamol, with an RR of 1.08 (95% CI 0.69 to 1.68, see Fig 3). No serious adverse events were reported, and for overall dropouts and dropouts due to adverse events, 95% CI overlapped the line of no difference. Thirteen patients treated with metamizole had cardiovascular adverse events, compared to two patients treated with paracetamol (RR = 3.48, 95% CI 1.07 to 11.27). All of these patients had hypotension due to intravenous injection of metamizole.

### Metamizole versus Aspirin

**Fig 4:**
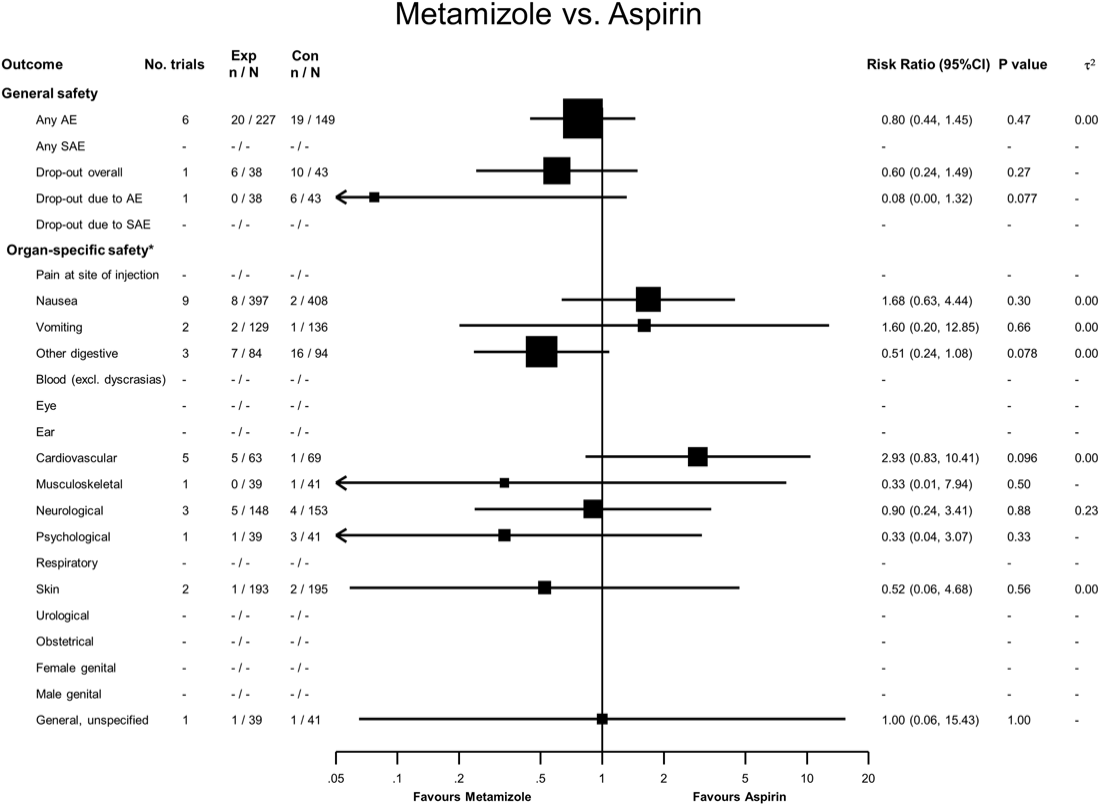
Forest plot—Metamizole versus Aspirin. Categories according to the International Classification of Primary Care (see Methods section and [13]). Results from single studies are of limited interpretability but are displayed for the sake of completeness. RR = risk ratio. AE = adverse events. SAE = serious adverse events.

Twenty adverse events were reported in 227 patients treated with metamizole, and 19 in 149 treated with aspirin, with a RR of 0.80 (95% CI 0.44 to 1.45, see Fig 4). No serious adverse events were reported, and for overall dropouts and dropouts due to adverse events, 95% CI overlapped the line of no difference. We found no statistically significant difference in organ-specific safety.

### Metamizole versus NSAIDs

**Fig 5:**
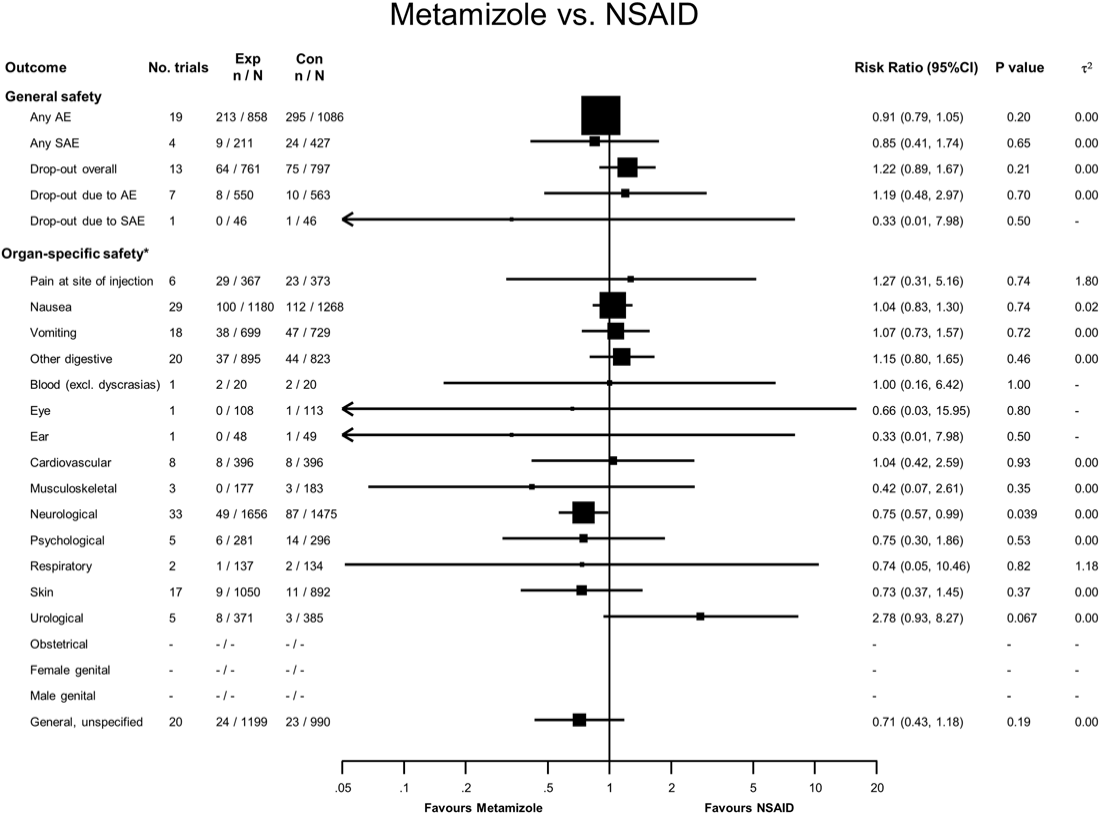
Forest plot—Metamizole versus NSAIDs. Categories according to the International Classification of Primary Care (see Methods section and [13]). Results from single studies are of limited interpretability but are displayed for the sake of completeness. RR = risk ratio. AE = adverse events. SAE = serious adverse events.

Two hundred and thirteen adverse events were reported in 858 patients treated with metamizole, compared to 295 adverse events in 1086 treated with NSAIDs, yielding a RR of 0.91 (95% CI 0.79 to 1.05, see Fig 5). Nine serious adverse events were reported in the metamizole group and 24 serious adverse events were reported in the NSAID group. With the exception of two postoperative hemorrhages after abdominoplasty described in participants allocated to an NSAID in one trial [56], the majority of the remaining 22 serious adverse events in two trials by the same research group were described as ‘cases of recurrence of renal pain that led to hospitalization [80,81]. For overall dropouts, dropouts due to adverse events and drop-outs due to serious adverse events, CI overlapped the line of no difference. One dropout due to serious adverse events was a post-operative hemorrhage in the NSAID group [56]. Forty-nine patients treated with metamizole had neurological adverse events compared to 87 treated with NSAIDs (RR 0.75, 95% CI 0.57 to 0.99). The most common reason was headache or unspecific vertigo and dizziness.

### Metamizole versus Opioids

**Fig 6:**
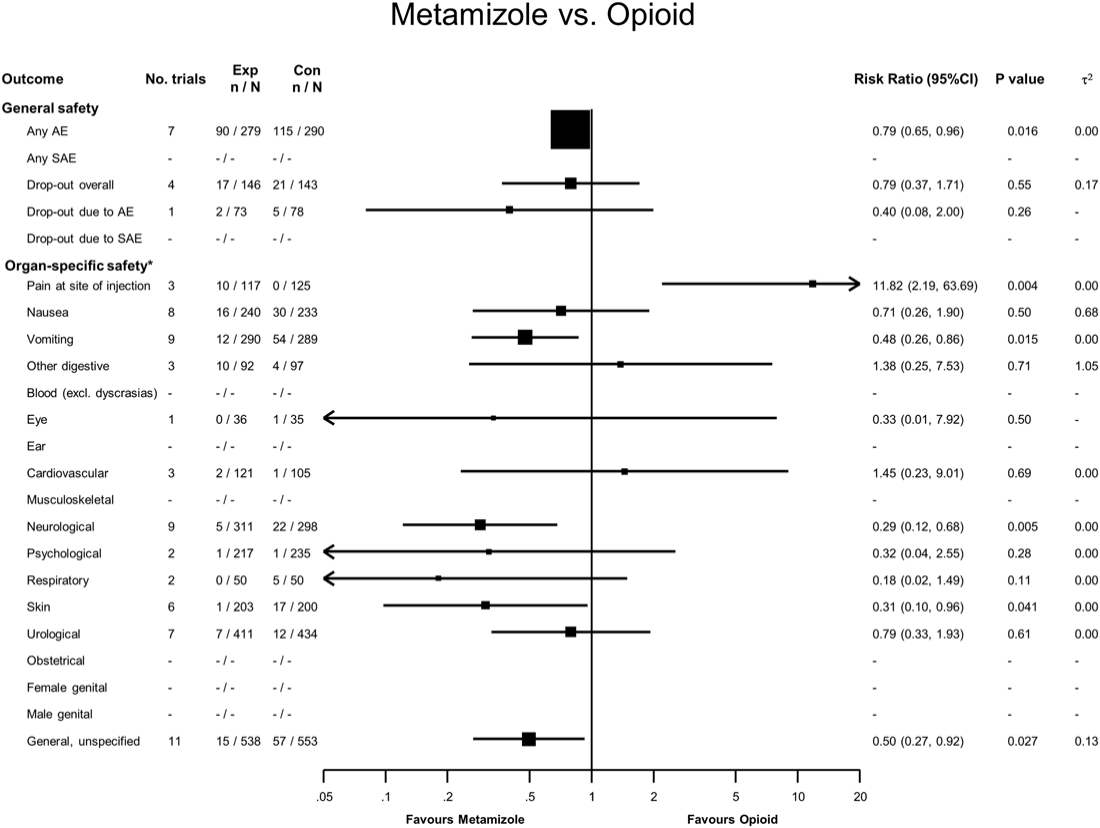
Forest plot—Metamizole versus Opioids. Categories according to the International Classification of Primary Care (see Methods section and [13]). Results from single studies are of limited interpretability but are displayed for the sake of completeness. RR = risk ratio. AE = adverse events. SAE = serious adverse events.

Ninety adverse events were reported in 279 patients treated with metamizole, compared to 115 adverse events in 290 treated with opioids, yielding a RR of 0.79 (95% CI 0.79 to 0.96, see Fig 6). No serious adverse events were reported, and for overall dropouts and dropouts due to adverse events, CI overlapped the line of no difference. Ten patients in the metamizole group reported pain at the injection site, compared to none in the opioid group (RR = 11.8, 95% CI 2.2 to 63.7). Twelve patients in the metamizole group reported vomiting compared to 54 in the opioid group (RR = 0.48, 95% CI 0.26 to 0.86), while five metamizole patients compared to 22 taking opioids reported neurological signs, most commonly vertigo, dizziness or headache (RR = 0.29, 95% CI 0.12 to 0.68). Unspecific and general complaints such as sweating, tiredness, somnolence, shaking and fever were less common in the metamizole group than in the opioid group (15 versus 57 events, RR = 0.50, 95% CI 0.27 to 0.92).

Tests of interaction indicated no evidence of an association between outcomes and dose or route of administration. Results of these interaction tests must be carefully interpreted given their low power, however.

### Serious adverse events, agranulocytosis and death

None of the trials reported agranulocytosis or withdrawals due to death. In addition, the trialists did not attribute any of the above-mentioned serious adverse events to metamizole.

## Discussion

In our meta-analysis of randomized controlled trials that compared the safety of metamizole to placebo and other analgesics, there was no difference in adverse events between metamizole and placebo, paracetamol, aspirin, or NSAIDs, and fewer adverse events compared to opioids. These 79 trials, which included almost 4000 patients with short-term metamizole use of less than two weeks, reported few serious adverse events, with no difference between metamizole and the comparators, and no cases of agranulocytosis.

Our approach to studying organ-specific differences identified several statistically significant differences in the frequency of adverse events, most of which seemed clinically plausible. First, compared to paracetamol, patients treated with metamizole had a significantly higher rate of adverse cardiovascular events, most commonly hypotension. Most of the trials in which these adverse events were reported used intravenously administered metamizole, which can decrease systolic blood pressure [96]. Second, compared with NSAIDs, metamizole was less likely to be associated with neurological adverse events such as headache, vertigo or dizziness. This finding is consistent with a standard textbook on drug side effects^1^, which describes neurological adverse events as “never” being caused by metamizole. Third, compared to opioids, metamizole was less frequently associated with neurological and unspecific adverse events, most commonly vertigo, dizziness, tiredness, sedation and CNS depression. Opioids and metamizole have different modes of action, and the described adverse events are well documented for opioids but not for metamizole [1]. However, it is not clear why metamizole caused more pain at the injection site compared to opioids.

Our systematic review was strengthened by the highly sensitive search strategy employed. We captured as many relevant studies as possible by including trials published in all languages, contacting the authors of studies published in the last 12 years and performing the additional reference list search. Because we included studies that examined metamizole for all indications, we were able to analyze 79 studies, while the three Cochrane reviews, which assessed clinical effectiveness only in postoperative pain, renal colic pain and acute primary headaches, analyzed 15 [2], 11 [3] and four [4] studies, respectively. We also included more patients; our study evaluated 8426 patients, while the Cochrane reviews evaluated 1436 [2], 1053 [3] and 636 [4]. Our search strategy enabled us to conduct a safety analysis and identify serious adverse events, which the Cochrane reviews were not able to do.

The generalizability of our results is limited by the methodological characteristics of the studies we included. Studies did not consistently assess adverse events, and only 17% of studies had a low risk of bias. Adverse events were also reported differently between trials, which further limits the validity of our results [97]. The trials we included were underpowered to demonstrate differences in drug safety. Few trials were conducted in ambulatory settings, limiting the generalizability of our results for community usage. The included trials also had very short follow-up periods, so it is impossible to draw conclusions about intermediate- and long-term adverse events from the available evidence. The small sample size and short-term follow-up of included trials means that the number of accumulated events (see Figs 2–6) is small for nearly all safety outcomes, which renders our results imprecise and our meta-analyses unreliable as previously discussed by Flather et al [98]. Because many of the included studies were conducted before mandatory trial registers were introduced, we cannot rule out publication bias; we did, however, search different trial registers to minimize the probability of bias.

We only considered the safety aspects of metamizole and did not evaluate efficacy, which the above-mentioned Cochrane reviews found was similar to that of other widely used analgesics for three different indications: postoperative pain, renal colic pain and acute primary headaches [2–4]. Given these findings, an analgesic should be chosen based on its safety profile. Metamizole was associated with fewer adverse events than opioids and had a better neurological side effect profile than NSAIDs. However, we found no differences regarding the most recently discussed end points of gastrointestinal and cardiovascular safety [10–12]. This contrasts with a comparative overall safety analysis of four different analgesics (aspirin, diclofenac, acetaminophen and metamizole) based on epidemiological studies published between 1970 and 1995 [7]. In this analysis, Andrade et al included data on short-term (one-week) use of the analgesics and estimated the excess mortality due to agranulocytosis, aplastic anemia, anaphylaxis and serious upper gastrointestinal complications. The authors found an excess mortality for metamizole of 25 per 100 million compared to 592 per million for diclofenac, 185 for aspirin and 20 for paracetamol. Serious upper gastrointestinal complications largely accounted for the excess mortality associated with diclofenac and aspirin [7].

None of the included randomized trials reported agranulocytosis, which is a rare but very harmful adverse event associated with metamizole [8]. There are huge variations in its estimated incidence [99], ranging from 1 case per 1431 prescriptions in a Swedish study [100] to 9 cases per million per year in the International Agranulocytosis and Aplastic Anemia Study [101]. Even if we assume the Swedish estimate to be correct, the number and size of the trials included in our review would be too small to yield more than only a few agranulocytosis cases. A systematic review of observational studies investigating the frequency of metamizole-associated agranulocytosis is currently under preparation by our working group.

## Conclusion

For short-term use in the hospital setting, such as to treat renal colic or postoperative pain, metamizole seems to be a safe choice when compared to other widely used analgesics. There is very limited information available on the intermediate- and long-term safety of metamizole. High-quality, adequately sized trials assessing metamizole-associated adverse events in the ambulatory setting are needed.

## Supporting Information

S1 Dataset Dataset. (XML)

S1 PRISMA Checklist (PDF)

S1 Table Search Strategy. (PDF)

S2 Table Definitions used to classify trials according to components of methodological quality. (PDF)

S3 Table Methodological characteristics of the trials. (PDF)

S4 Table Label of variables used in S1 Dataset. (PDF)
