# Supplementary material for: Metamizole-Associated Adverse Events: A Systematic Review and Meta-Analysis
Source: PLoS One. 2015 Apr 13;10(4):e0122918. doi: 10.1371/journal.pone.0122918 (PMC4405027; doi:10.1371/journal.pone.0122918)
Supplement: S1 Table — (PDF) [file pone.0122918.s003.pdf]

Supplement 1: Search strategy

**Search terms for metamizole**	29. metamizol$.mp
01. analginum.mp	30. methampyron$.mp
02. algi.mp	31. methanesulphonat$.mp
03. anador.mp	32. methylmelubrin$.mp
04. analgin§.mp	33. (neo-melubrina).mp
05. analgit.mp	34. (Neuro Fortamin).mp
06. baralgin.mp	35. nolotil§.mp
07. berlosin.mp	36. noramidopyrin$.mp
08. conmel.mp	37. noraminophenazon$.mp
09. dalmasin$.mp	38. norgesic.mp
10. debela.mp	39. novalgin$.mp
11. dipimax.mp	40. novamidazofen.mp
12. dipirona.mp	41. novaminsulfon$.mp
13. dipyrone/	42. optalgin$.mp
14. dipyrone.mp	43. phanalgin$.mp
15. dolgan.mp	44. pirazol$.mp
16. dornal .mp	45. pirenil.mp
17. dorona.mp	46. prodolina.mp
18. doran.mp	47. pyranol$.mp

19. exalgin$.mp	48. pyrethan$.mp
20. inalgon$.mp	49. pyril.mp
21. lasain.mp	50. pyron$.mp
22. magnol.mp	51. (Spasmo Inalgon).mp
23. magnopyrol.mp	52. sulpyrin$.mp
24. maxiliv.mp	53. suprim.mp
25. metilon.mp	54. terminal.mp
26. minalgin.mp	55. (V-Talgin).mp
27. mineral.mp	56. vegal.mp
28. melubrina.mp	57. zolidin$.mp
