# Supplementary material for: Metamizole-Associated Adverse Events: A Systematic Review and Meta-Analysis
Source: PLoS One. 2015 Apr 13;10(4):e0122918. doi: 10.1371/journal.pone.0122918 (PMC4405027; doi:10.1371/journal.pone.0122918)
Supplement: S2 Table — (PDF) [file pone.0122918.s004.pdf]

Supplement 2

Definitions used to classify trials according to components of methodological quality

Item	***Definition***
Randomization	We considered randomization adequate, if it was done using random number tables, computer-generated random numbers, minimization, coin tossing, shuffling cards, throwing dice, drawing of lots or other “simple randomization“ techniques.
Concealment of allocation	We considered concealment of allocation adequate, if it was done using central allocation, sequentially numbered, opaque, sealed envelopes, sequentially numbered drug containers of identical appearance.
Blinding of patients	We considered blinding of patients adequate, if it was described as “double blind“.
Adverse event assessment	We considered adverse event assessment adequate, if it was done in a prospective and systematic way.
Dropouts / Exclusions	We considered the risk of bias from dropouts / exclusions as “low” if - in case of a trial duration ≤48 hours - the dropout rate was ≤10% per group and the reasons for dropping out were reported and if – in case of a trial duration > 48 hours – the dropout rate was

	≤20% per group and the reasons for dropping out were reported and an intention-to-treat analysis was conducted.
Incomplete adverse event assessment	We considered adverse event assessment as “complete”, if all patients were assessed for adverse events.
Analyses	We considered statistical analyses to be adequate if all randomized patients were included in the analysis according to the intention-to-treat principle.
