# Supplementary material for: Metamizole-Associated Adverse Events: A Systematic Review and Meta-Analysis
Source: PLoS One. 2015 Apr 13;10(4):e0122918. doi: 10.1371/journal.pone.0122918 (PMC4405027; doi:10.1371/journal.pone.0122918)
Supplement: S3 Table — (PDF) [file pone.0122918.s005.pdf]

Supplement 4

Methodological characteristics of the trials

Trial	Randomization	Concealment	Blinding	Adverse events assessment	Dropouts / Exclusion	Incomplete adverse events assessment	Analyses
**Ajgaonkar**	unclear	unclear	adequate	unclear	unclear	unclear	no
**Arnau**	adequate	unclear	adequate	no	adequate	adequate	no
**Atalay**	unclear	unclear	no	no	adequate	adequate	adequate
**Babej-Dölle**	adequate	adequate	no	no	adequate	adequate	adequate
**Bagan**	unclear	unclear	adequate	no	unclear	adequate	no
**Bigal**	adequate	unclear	adequate	unclear	adequate	unclear	no
**Bilgin**	unclear	unclear	no	unclear	unclear	unclear	no
**Blendinger**	unclear	unclear	no	no	adequate	adequate	adequate

**Bloch**	unclear	adequate	adequate	no	no	adequate	adequate
**Boraks**	unclear	unclear	adequate	unclear	unclear	unclear	no
**Braun**	unclear	unclear	adequate	adequate	unclear	adequate	no
**Brodner**	adequate	adequate	adequate	adequate	adequate	adequate	adequate
**Castro**	unclear	unclear	adequate	unclear	unclear	no	no
**Castro Gonzales**	unclear	unclear	adequate	unclear	unclear	no	no
**Cruz**	unclear	unclear	no	unclear	adequate	adequate	adequate
**De Miguel Rivero**	unclear	adequate	adequate	no	adequate	adequate	no
**Diaz-Chavez**	adequate	unclear	no	unclear	unclear	unclear	no
**Dos Santos Pereira**	unclear	unclear	no	unclear	adequate	adequate	adequate
**Duarte Souza**	unclear	unclear	adequate	no	adequate	adequate	adequate
**Fernandes Filho**	unclear	unclear	adequate	unclear	adequate	adequate	adequate

**Ferrario**	unclear	unclear	no	unclear	adequate	adequate	no
**Gomes-Marquez**	adequate	unclear	no	unclear	no	no	no
**Gonzalez-Garcia**	unclear	unclear	adequate	no	unclear	adequate	adequate
**Grundmann**	adequate	adequate	adequate	adequate	adequate	adequate	adequate
**Guberti**	unclear	unclear	no	unclear	adequate	unclear	no
**Hernandez Llenas**	unclear	unclear	no	unclear	adequate	adequate	adequate
**Herrera Barroso**	unclear	adequate	adequate	unclear	adequate	adequate	adequate
**Ibarra-Ibarra**	unclear	unclear	adequate	unclear	adequate	adequate	adequate
**Jage**	unclear	unclear	no	no	adequate	adequate	adequate
**Jovic**	unclear	unclear	no	no	adequate	adequate	adequate
**Kampe**	adequate	adequate	adequate	adequate	adequate	adequate	adequate
**Karaman**	unclear	adequate	adequate	adequate	adequate	adequate	adequate

**Kemal**	unclear	unclear	no	unclear	unclear	unclear	no
**Knüsel**	unclear	unclear	adequate	no	no	no	no
**Krymchantowski**	no	unclear	no	no	adequate	adequate	adequate
**Landwehr**	adequate	adequate	adequate	adequate	unclear	no	no
**Lehmann**	adequate	unclear	adequate	unclear	adequate	adequate	adequate
**Lehtonen**	unclear	unclear	no	no	adequate	adequate	adequate
**Marin-Bertolin**	unclear	unclear	adequate	no	unclear	adequate	no
**Martin-Duce**	unclear	unclear	adequate	no	unclear	no	no
**Martinez-Martin**	adequate	unclear	adequate	adequate	no	adequate	no
**Mateu**	unclear	unclear	adequate	unclear	unclear	no	no
**Mehta**	unclear	unclear	adequate	unclear	unclear	no	no
**Monso**	unclear	unclear	adequate	no	adequate	adequate	adequate

**Muriel**	unclear	unclear	adequate	no	unclear	unclear	no
**Muriel-Villoria**	unclear	unclear	adequate	no	unclear	unclear	no
**Ocampo Flores**	unclear	unclear	adequate	unclear	no	no	no
**Pardo**	unclear	unclear	no	unclear	adequate	adequate	adequate
**Patel**	unclear	unclear	adequate	unclear	unclear	unclear	no
**Pavlik**	adequate	unclear	adequate	no	adequate	adequate	adequate
**Peiró**	adequate	unclear	no	no	unclear	unclear	no
**Pernia**	unclear	unclear	adequate	unclear	adequate	adequate	adequate
**Pinto**	unclear	unclear	adequate	unclear	adequate	adequate	adequate
**Planas**	adequate	unclear	adequate	no	unclear	no	no
**Prada**	unclear	no	adequate	unclear	adequate	unclear	no
**Primus**	unclear	no	no	no	unclear	unclear	no

**Rawal**	adequate	adequate	adequate	adequate	no	adequate	adequate
**Rejman**	unclear	unclear	no	unclear	adequate	adequate	adequate
**Reyes**	adequate	unclear	adequate	no	adequate	adequate	adequate
**Reyes-Armijo**	unclear	unclear	adequate	unclear	adequate	unclear	adequate
**Rodriguez**	unclear	unclear	adequate	adequate	unclear	unclear	no
**Rubinstein**	unclear	unclear	adequate	unclear	adequate	adequate	adequate
**Sanchez-Carpena 2003**	adequate	unclear	adequate	no	adequate	adequate	adequate
**Sanchez-Carpena 2007**	adequate	adequate	adequate	no	adequate	adequate	adequate
**Saray**	unclear	adequate	adequate	adequate	unclear	adequate	no
**Savoca**	unclear	unclear	no	unclear	adequate	unclear	no
**Schmieder**	unclear	unclear	no	no	unclear	adequate	no

**Sener**	adequate	unclear	adequate	unclear	unclear	adequate	adequate
**Spacek**	adequate	adequate	adequate	adequate	adequate	adequate	adequate
**Stankov 1994**	unclear	unclear	adequate	no	unclear	adequate	no
**Stankov 1995**	adequate	unclear	no	no	adequate	unclear	adequate
**Steffen**	adequate	unclear	adequate	no	adequate	adequate	adequate
**Striebel**	unclear	unclear	adequate	no	unclear	unclear	no
**Tempel**	unclear	adequate	adequate	no	adequate	adequate	adequate
**Tonolli Jacob**	unclear	unclear	adequate	unclear	adequate	adequate	adequate
**Torres 1993**	unclear	unclear	adequate	adequate	adequate	adequate	adequate
**Torres 2001**	adequate	unclear	adequate	no	adequate	unclear	adequate
**Uzun**	adequate	unclear	adequate	adequate	adequate	adequate	adequate
**Vargha von Szeged**	unclear	unclear	no	no	adequate	adequate	adequate
