# Supplementary material for: Metamizole-Associated Adverse Events: A Systematic Review and Meta-Analysis
Source: PLoS One. 2015 Apr 13;10(4):e0122918. doi: 10.1371/journal.pone.0122918 (PMC4405027; doi:10.1371/journal.pone.0122918)
Supplement: S4 Table — (PDF) [file pone.0122918.s006.pdf]

Supplement 5

Label of variables used in Supplement 6 (Dataset of all outcomes provided for each study separately)

The variable names should be interpreted as following:

"yes" concerns the number of individuals that experienced the event;
"no" concerns the number of individuals that did not experience the event;

the number that comes at the end of variable names indicate the intervention:

(1) Metamizole

(2) Placebo

(3) Aspirin

(4) NSAID

(5) Opioid

(6) Paracetamol

AE: adverse event; SAE serious adverse event.
